# Editorial: What we aim for

**DOI:** 10.1093/emph/eov008

**Published:** 2015-04-21

**Authors:** Stephen C. Stearns

**Affiliations:** Editor-in-Chief, Evolution, Medicine, and Public Health, New Haven, CT

What is the level of quality and impact that we aim for in articles published in *EMPH*? This is an interdisciplinary journal that addresses a broad audience. Interdisciplinary work is often thought to suffer from a lack of rigor because it can escape the scrutiny that referees habitually apply to work in core disciplines. That is not what we intend here. We hold interdisciplinary work to a high standard: it should meet the requirements for publication in the top journals of all the disciplines on which it draws, both evolutionary and biomedical. We want original research that creatively deepens and extends our understanding of the power of evolutionary thought to explain biomedical science. We want critical reviews that build on their summary of current understanding to point to gaps in our knowledge and to research to be done. And we want commentaries that thoroughly establish the plausibility of the exciting hypotheses that they suggest.

*EMPH* provides a novel venue and an interested audience specifically focused on the added value that evolutionary insights bring to medicine and public health. This is the best place to publish work aimed at that international community.

While it is completely understandable that authors seek to publish in established high-impact journals, those journals do not accept all articles that are worthy of them. *EMPH* provides a destination for some of the excellent articles that do not make it into the highest impact journals.

What sort of work should not be submitted to *EMPH*? We are not interested in empirical articles that simply repeat observations already well established, in review articles that simply summarize the literature, or in commentaries that advance speculative evolutionary ideas not grounded in rigorous theory or solid evidence.

The criteria we apply in reviewing Clinical Briefs deserve special mention, for this is a new category of publication. The format is short and highly structured, and the topic requires careful thought. Clinical Briefs are not the place to speculate, and they are not the place to report on controversies in progress. They should represent conclusions that are broadly agreed on and reliable, that are based on evolutionary insights that give added value, and that have clinical applications.

Although it is not easy to start a new journal in a new interdisciplinary field, starting one that is not rigorous is not worth doing at all. We are off to a good start. We will continue to publish interesting work that is rigorous and reliable. It is well worth the effort to keep the bar high.

